# Intrapericardial Administration of Secretomes from Menstrual Blood-Derived Mesenchymal Stromal Cells: Effects on Immune-Related Genes in a Porcine Model of Myocardial Infarction

**DOI:** 10.3390/biomedicines10051117

**Published:** 2022-05-11

**Authors:** María Ángeles de Pedro, María Pulido, Federica Marinaro, Verónica Álvarez, Claudia Báez-Díaz, Virginia Blanco, Juan Carlos Silla-Castro, Fátima Sanchez-Cabo, Francisco Miguel Sánchez-Margallo, Verónica Crisóstomo, Javier G. Casado, Esther López

**Affiliations:** 1 Stem Cell Therapy Unit, Jesús Usón Minimally Invasive Surgery Centre, Carretera Nacional 521, Km 41.8-10071, 10004 Cáceres, Spain; madepedro@ccmijesususon.com (M.Á.d.P.); mpulido@ccmijesususon.com (M.P.); fmarinaro@ccmijesususon.com (F.M.); valvarez@ccmijesususon.com (V.Á.); cbaez@ccmijesususon.com (C.B.-D.); vblanco@ccmijesususon.com (V.B.); crisosto@ccmijesususon.com (V.C.); elopez@ccmijesususon.com (E.L.); 2 CIBER de Enfermedades Cardiovasculares (CIBERCV), C. de Melchor Fernández Almagro, 3, 28029 Madrid, Spain; 3 Bioinformatics Unit, Centro Nacional de Investigaciones Cardiovasculares, C. de Melchor Fernández Almagro, 3, 28029 Madrid, Spain; juancarlos.silla@cnic.es (J.C.S.-C.); fscabo@cnic.es (F.S.-C.); 4 Immunology Unit, University of Extremadura, Campus Universitario, Av. de la Universidad, s/n, 10003 Cáceres, Spain; 5 Institute of Molecular Pathology Biomarkers, University of Extremadura, 10003 Cáceres, Spain

**Keywords:** immune response, mesenchymal stromal cells (MSCs), myocardial infarction, secretome, swine

## Abstract

Acute myocardial infarction (AMI) is a manifestation of ischemic heart disease where the immune system plays an important role in the re-establishment of homeostasis. We hypothesize that the anti-inflammatory activity of secretomes from menstrual blood-derived mesenchymal stromal cells (S-MenSCs) and IFNγ/TNFα-primed MenSCs (S-MenSCs*) may be considered a therapeutic option for the treatment of AMI. To assess this hypothesis, we have evaluated the effect of S-MenSCs and S-MenSCs* on cardiac function parameters and the involvement of immune-related genes using a porcine model of AMI. Twelve pigs were randomly divided into three biogroups: AMI/Placebo, AMI/S-MenSCs, and AMI/S-MenSCs*. AMI models were generated using a closed chest coronary occlusion-reperfusion procedure and, after 72 h, the different treatments were intrapericardially administered. Cardiac function parameters were monitored by magnetic resonance imaging before and 7 days post-therapy. Transcriptomic analyses in the infarcted tissue identified 571 transcripts associated with the Gene Ontology term *Immune response*, of which 57 were differentially expressed when different biogroups were compared. Moreover, a prediction of the interactions between differentially expressed genes (DEGs) and miRNAs from secretomes revealed that some DEGs in the infarction area, such as *STAT3, IGFR1*, or *BCL6* could be targeted by previously identified miRNAs in secretomes from MenSCs. In conclusion, the intrapericardial administration of secretome early after infarction has a significant impact on the expression of immune-related genes in the infarcted myocardium. This confirms the immunomodulatory potential of intrapericardially delivered secretomes and opens new therapeutic perspectives in myocardial infarction treatment.

## 1. Introduction

Ischemic heart disease has been classified by World Health Organization as the major cause of death worldwide in 2019 (https://www.who.int/news-room/fact-sheets/detail/the-top-10-causes-of-death, accessed on 30 March 2022). This disease is frequently associated with acute myocardial infarction (AMI), whose molecular mechanisms have been widely studied. AMI is produced by the occlusion of the coronary artery, that leads to myocardial tissue damage. Myocardial reperfusion injury activates a wide range of cellular and molecular processes including inflammation, neutrophil infiltration, oxidative stress, intracellular calcium overload, pH alteration, mitochondrial permeability increase and, consequently, apoptosis [1].

Inflammation has a key role in the clearance of cellular debris and the restoration of homeostasis in the infarct zone. This mechanism is initiated by the release of “danger signals” termed DAMPS (danger-associated molecular patterns) from necrotic cardiomyocytes to the extracellular space. These signals are recognized by the surrounding cardiomyocytes and fibroblasts, activating the secretion of proinflammatory cytokines and chemokines involved in the recruitment of inflammatory cells to the infarct area. Neutrophils first, followed by monocytes and leukocytes, migrate to the injury site releasing proinflammatory cytokines and initiating the phagocytosis of dead cells. The “inflammatory phase” is followed by the “proliferative phase”, where macrophages resolve the initial inflammatory reaction through the release of anti-inflammatory, profibrotic, and angiogenic factors such as interleukin (IL) 10, transforming growth factor (TGF) β, or vascular endothelial growth factor (VEGF). This new anti-inflammatory milieu leads to the removal of inflammatory cells. This phase continues with the formation of granulation tissue and collagen-rich scar formation, completing the process of infarct healing [2].

Regarding the molecular mechanisms underlying myocardial infarction, an important scientific advance has been achieved by the development of “omics” techniques. This methodology, together with the use of clinically relevant animal models, has allowed a deep knowledge of AMI progression. In this sense, the porcine model has been widely used as an experimental animal to study the pathophysiology of acute or chronic myocardial infarction. In terms of size, weight, anatomy, and physiology, the porcine heart is very similar to the human heart [3], and surgical procedures, such as permanent coronary occlusion [4], percutaneous embolization [5], or endovascular approaches [6,7] have been used to induce myocardial infarction.

Previous studies in murine models using proteomic and transcriptomic analyses have confirmed the involvement of the immune system in different stages of AMI [8]. In the case of large animal models, such as the pig, the characterization of molecular changes in the infarcted area have revealed the implication of apoptosis-related genes [9] or proteins involved in acute-phase response signaling, response to wounding, and production of nitric oxide and reactive oxygen species [10].

It is interesting to note that, although the molecular characterization of AMI has been sufficiently investigated, the molecular events that occur when testing different treatments in large animal models, including mesenchymal stromal cell (MSCs) therapy, have not been fully characterized. As an example, secretomes-derived therapies for AMI have been considered a novel therapeutic product due to their complex composition, consisting of extracellular vesicles (EVs), RNA, soluble factors, etc. The biological composition of these secretomes seems to be a very effective tool to modulate the altered molecular mechanisms in AMI, promoting angiogenesis, cardiac regeneration [11], and exacerbated inflammatory response [12]. However, despite the promising results using extracellular vesicles in clinically relevant animal models, a complete characterization of the infarcted and treated tissues is still necessary.

Here, menstrual blood stromal cells (MenSCs) have been selected to evaluate their effectiveness as a potential treatment. Menstrual blood is a novel source of MSCs that stands out from other MSCs because of its ease of regular collection, non-invasiveness, and lack of ethical concerns. In addition to having a high proliferation rate and low immunogenicity, it remains stable without mutations in vitro for at least 20 passes without losing its biological properties [13]. Both MenSCs [14] and their extracellular vesicles [15] have already demonstrated to be effective in the improvement of cardiac function in infarcted rat hearts.

Therefore, in this study, we have evaluated the therapeutic effect of secretome concentrated from MenSCs (S-MenSCs) in a porcine model of AMI. Our previous studies about these secretomes demonstrated that TGFβ was involved in the regulation of T CD4+ cells [16] and that their immunomodulatory activity could be enhanced by priming of precursor cells with interferon (IFN) γ [17] and the combination of IFNγ and tumor necrosis factor (TNF) α [18]. Based on that, the main objective of this study was evaluating the effect of MenSCs and secretomes released by IFNγ and TNFα-primed MenSCs (hereinafter, “S-MenSCs*”) on immune-related genes. This analysis was performed in the infarcted area (left ventricle) of a porcine model. Our results using transcriptomic sequencing and in silico analyses using bioinformatic tools demonstrated the differential expression of immune-related genes in infarcted myocardium treated with S-MenSCs or S-MenSCs*. These changes in profile expression demonstrate the immunomodulatory capacity of secretomes under myocardial inflammatory conditions.

## 2. Results

### 2.1. Effects of S-MenSCs and S-MenSCs* Administration on Cardiac Function

**Figure 1:**
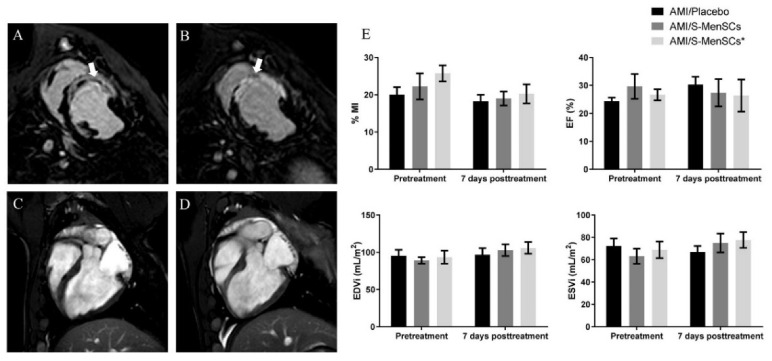
Cardiac function parameters. Myocardial infarction percentage (%MI), ejection fraction (EF), end-diastolic volume (EDV), and end-systolic volumes (ESV) were evaluated by cardiac magnetic resonance just before treatment administration and 7 days after therapy in all biogroups. (**A**–**D**) Representative images for A and B delayed enhancement, short-axis view before treatment (**A**) and after one week (**B**) were obtained. The infarcted area is indicated by white arrows. Dark areas within the hyperenhancement represent microvascular obstruction, typical of the early stages of the model. Four Chamber views obtained before treatment (**C**) and at one week (**D**). All images correspond to the same animal. (**E**) Bar graphs represent the mean of each parameter (±standard error) before and after treatment.

Cardiovascular magnetic resonance imaging was performed at day 3 post-infarction, just before treatment administration (pretreatment), and at day 7 post-therapy (Figure 1A–D). These time points allowed us to determine the early effects of S-MenSCs and S-MenSCs* on cardiac function parameters. The percentage of myocardial infarcted area (%MI), expressed as a percentage of the estimated infarcted size dividing by entire area of left ventricle, was reduced after both therapies, but with no significant differences inter- and intra-groups. Regarding cardiac hemodynamic parameters, ejection fraction (EF), end-diastolic volume (EDV), and end-systolic volumes (ESV) were calculated. Our results did not show any significant alterations in these parameters and data remained fairly stable both before and 7 days after therapy in all biogroups (Figure 1E).

### 2.2. DEGs Identified and Related to the Immune System Process

**Figure 2:**
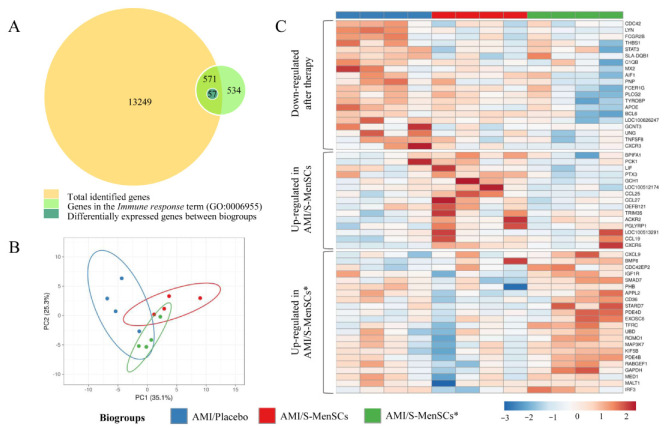
DEGs in infarcted tissue related with *Immune response* term (GO:0006955). (**A**) The BioVenn diagram represents the overlap between different gene subsets. The yellow circle represents total genes identified in cardiac tissue by microarray data analysis; the green circle represents genes associated to the *Immune response* term (GO:0006955) in *Sus scrofa* species; the blue circle represents all DEGs between biogroups, which are clustered in the Immune response term. (**B**) PCA analysis of all DEGs in cardiac tissue related to the *Immune response* term. Unit variance scaling is applied to rows; singular value decomposition (SVD) with imputation is used to calculate principal components. X and Y axis show principal component 1 and principal component 2 that explain 25.3% and 35.1% of the total variance, respectively. Prediction ellipses are such that with a probability of 0.95, a new observation from the same group will fall inside the ellipse. (**C**) The heatmap shows different expressions of all DEGs, and it is divided into three clusters: down-regulated after therapy (S-MenSCs or S-MenSCs* administration), up-regulated in AMI/S-MenSCs, and up-regulated in AMI/S-MenSCs*. DEGs: differentially expressed genes with *p* value < 0.05 without FDR optimization.

The gene expression profile of the infarcted area was analyzed. Expression data obtained from microarray data analysis identified a total of 13,820 transcripts annotated as porcine genes. All these genes were then classified according to Gene Ontology term annotation and our analysis was focused on those genes associated with the “*Immune response*” term (GO:0006955). Our results demonstrated that a total of 571 genes were classified into the term “*Immune response*”. Expression data normalization allowed us to analyze the differential expression between the transcripts of infarcted tissue from different biogroups. The statistical comparisons (without FDR correction) of transcripts in the infarcted areas, demonstrated that 57 transcripts were differentially expressed (*p* value < 0.05) when comparing the different biogroups (Figure 2A).

The 57 differentially expressed transcripts were subjected to PCA. This analysis allowed us to increase the interpretability of the results. According to PCA, the transcripts corresponding to different biogroups revealed a certain degree of divergence between groups (Figure 2B).

These transcripts were represented in a heatmap plotted in three different clusters: down-regulated after S-MenSCs or S-MenSCs* administration, up-regulated in AMI/S-MenSCs, and up-regulated in AMI/S-MenSCs* (Figure 2C).

### 2.3. DEGs and Predicted Interactions with miRNAs in Secretomes

Once the DEGs in infarcted tissues were identified and considering that secretomes contain a wide range of miRNAs, we aimed to identify hypothetical interactions between the 10 most abundant miRNA identified in S-MenSCs and S-MenSCs* (Table S1).

Experimentally validated miRNA target genes were filtered by “strong evidence” using miRTargetLink. Unfortunately, hsa-miR-12136 was not found in the database of miRTargetLink, so the analysis was performed with nine of the top ten most abundant miRNAs in S-MenSCs*. According to this analysis, 367 genes were targeted by the top ten abundant miRNAs from S-MenSCs, and 440 genes were targeted by the top ten abundant miRNAs from S-MenSCs* (data not shown).

**Figure 3:**
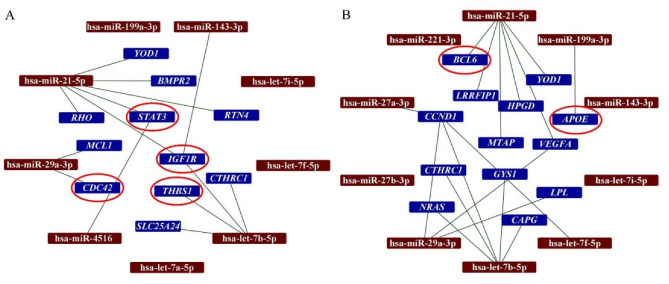
Networks between miRNAs in secretomes and DEGs in cardiac tissue. The miRTargetLink analysis was performed on the ten top-abundant expression miRNAs in S-MenSCs (**A**) and S-MenSCs* (**B**). Interaction networks between miRNAs and target genes that are differentially expressed in AMI/S-MenSCs (**A**) and AMI/S-MenSCs* (**B**) infarcted tissues. The red circles represent DEGs related to the *Immune System* GO term. DEGs: differentially expressed genes with *p* value < 0.05 without FDR optimization.

In this study, we have analyzed the interactions between the top ten most abundant miRNAs in secretomes with the DEGs in the infarcted tissue. Our analysis demonstrated that hsa-miR-21-5p interacts with 6 DEGs detected when AMI/Placebo group and AMI/S-MenSCs group were compared. Indeed, two of these genes, *STAT3* and *IGF1R*, were classified in the “*Immune system*” GO term (Figure 3A). Additionally, the differentially expressed gene *BCL6,* detected when AMI/Placebo and AMI/S-MenSCs* groups were compared, was found to be targeted by hsa-miR-21-5p (Figure 3B).

It is important to note that the miRTargetlink analysis also revealed interactions between *IGF1R* (DEG when AMI/Placebo and AMI/S-MenSCs groups were compared) and the three top abundant miRNAs from S-MenSCs: hsa-miR-143-3p, hsa-miR-21-5p, and hsa-let-7b-5p.

Based on orthologous genes theory [19] and using g:Orth tool every targeted gene was identified like orthologous gene in pig (data not shown).

### 2.4. Validation of Pre-Selected Transcripts

**Figure 4:**
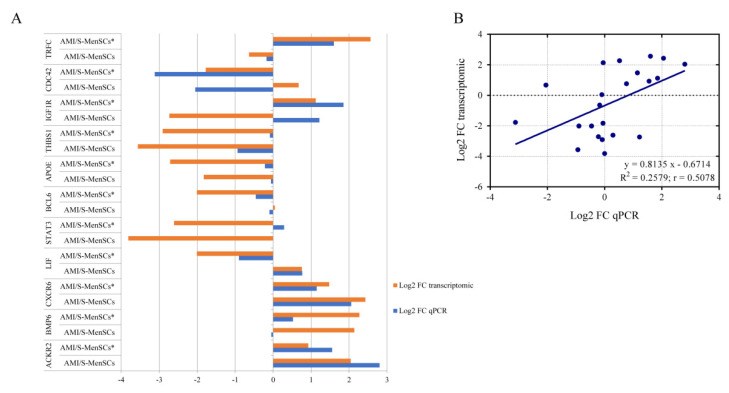
Validation of microarray results by qPCR. (**A**) The column bar graph represents the fold change expression of eleven pre-selected genes in infarcted tissue assessed by microarray data analysis (orange bars) and by qPCR experiments (blue bars). (**B**) The scatter plot shows the fold change correlation between transcriptomic results obtained by microarray analysis and qPCR results of eleven pre-selected genes. Microarray and qPCR data were log-transformed and blue line represents lineal regression. R^2^ = coefficient of determination; r = Pearson correlation coefficient.

qPCR was used to validate the gene expression profiles of eleven DEGs from the previous microarray results. As shown in Figure 4, in AMI/S-MenSCs*, 17 out of 22 sample analyzed showed congruent results between transcriptomic and qPCR results. However, *BCL6, STAT3, IGFR1, CDC42* and *BMP6* expressions were not confirmed by qPCR only in one of the biogroups (Figure 4A).

In general, a high consensus in fold changes was observed between qPCR and microarray data. Moreover, this trend was verified by linear regression analysis obtaining a significant Pearson correlation coefficient (r) (r = 0.5078, *p* value = 0.0158) (Figure 4B), thus validating the microarray results.

## 3. Discussion

A growing scientific interest has been focused on the use of secretome-derived products as therapeutic agents like isolated EVs or exosomes. Their ability to transport and transfer bioactive compounds makes them a suitable option for the treatment of very different disorders. The choice of secretome precursor cells is important because their origin determines their content and usefulness.

Based on that, we aimed to determine the therapeutic effect of these secretomes (primed or not) in a clinically relevant animal model of AMI. Moreover, considering the immunomodulatory profile of these secretomes, our interest was focused on immune-related genes expressed in the infarcted areas. It is important to note that tissue sampling was not uniquely restricted to the infarcted area, and the gene expression will also be analyzed in the remote regions of myocardium infarction. Taking into account that the administered secretomes could be distributed in the entire pericardial fluid, and that remote myocardium is directly related to left ventricular remodeling [20], our studies are currently focused on gene expression analyses in remote tissues.

Our in vivo experimental design started with a standardized endovascular model of balloon occlusion, which exhibits great similarities with the onset of AMI in humans [21]. The intrapericardial administration was performed at day 3 post-infarction. This “resting period” was necessary to ensure the animal’s survival and recovery for the subsequent surgical procedures. Moreover, according to our preclinical studies, 7 days represent an optimal timepoint to identify immunological changes and alterations of biochemical parameters in peripheral blood following the administration of the treatment [22]. Although, hematological and biochemical parameters were also analyzed in this study (data not shown), but the small sample size and the high experimental variability between animals made it difficult to obtain significant results in these unspecific parameters.

It is important to emphasize that an innovative aspect of this study is the administration route for the delivery of secretomes, which is different from previous studies using intravenous, intramyocardial, or intracoronary approaches [23,24,25]. The intrapericardial delivery of adult stem cells and EVs was previously assessed in a porcine model and was found to be safe for MSCs and cardiosphere-derived cell (CDCs) administrations [26,27]. Moreover, our group has recently demonstrated that the intrapericardial administration of EVs from CDCs triggered an M2 polarization during the acute phase of myocardial infarction [12]. In addition to being a safe and feasible access route, the intrapericardial delivery allows the diffusion of the administered therapy from epicardium to the endocardium, the most affected layer in ischemia/reperfusion injury [28].

In this study, we aimed to determine the therapeutic effect of S-MenSCs in terms of cardiac function parameters using cardiovascular magnetic resonance and gene expression analysis by microarrays. Cardiac function parameters and the expression of immune-related genes in the infarcted areas were analyzed at 7 days post-administration.

In our first set of data, although the administrations of therapies seemed to slightly improve cardiac function parameters, especially infarction size, these results were not statistically corroborated, maybe due to the small sample size (restricted by the ethical committee on animal experiments). Another limitation could be the occurrence of edema, hemorrhage, and inflammation which persists in the myocardium 7–10 days after reperfusion and can contribute to the overestimation of myocardial infarction size and underestimation of blood flow [29]. Moreover, the timeline of the present study may partly be the cause of the absence of significant changes to cardiac function parameters. Different time points for intrapericardial administrations and different time points for euthanasia may contribute to more significant results. In this sense, it is well known that the inflammatory phase starts a few hours after reperfusion and for this reason, we cannot discard that greater effects of secretomes could be observed in the early stages. Similarly, infarct healing mechanisms may still be ongoing at later time points, so cardiac magnetic resonance at 10 to 30 days may result in the improvement of cardiac function parameters. Additionally, it is important to note that, although swine models are widely used for cardiovascular disease research, the use of healthy young animals could limit the extrapolability of these results. In this context, and although the trend is very optimistic, these preliminary results require further investigation.

Regarding the transcriptomic profiling of the infarcted areas, our methodological approach with porcine microarrays allowed us to analyze thousands of genes and to compare their expression under different treatments. In our previous study, S-MenSCs treatment content and secretome from MenSCs prime with IFNγ were deeply characterized by NGS and proteomic analysis, revealing that most of the significant differences after prime cells were observed on immune-related proteins [18]. Considering the immunomodulatory capacity of these secretomes [16,17], our analysis was filtered by Gene Ontology and restricted to 571 genes classified into the “Immune Response” GO term. This analysis excludes thousands of genes involved in many different biological processes and pathways, which may have a key role in myocardial infarction [30]. Accepting that our analysis was not all-inclusive and strictly limited to immune response-related genes, here we could identify differentially expressed transcripts among the different groups.

The genes classified in the first cluster of the heatmap resulted to be down-regulated after both treatments, regardless of the use of basal or primed secretomes. Although the secretome content is supposed to be different between primed and basal MenSCs, a lot of mechanisms remained unaltered after priming cells with TNFα and INFγ. Although some of the differentially expressed genes may deserve a proper discussion, we will only focus on two of them. For example, a decrease of CXCR3 in the infarcted areas of treated animals was observed. This molecule is a chemokine receptor that binds inflammatory chemokines (CXCL9, CXCL10, CXCL11) and it was found to be expressed in immune and endothelial cells being involved in the homing of Th1 cells [31,32,33]. Moreover, thrombospondin-1, the THBS1 gene product, is a ligand of the immunoregulatory receptor CD47. A decrease in the expression could reduce the transendothelial migration of neutrophils, monocytes, and other leukocytes in the infarcted myocardium as well as the lymphocyte response [34]. Both genes participate in the recruitment of inflammatory cells to the infarction site, so their downregulation may reduce the excessive recruitment of leukocytes and adverse inflammatory reactions [35]. Similarly, both genes are related to angiogenesis, where their down-regulation improves angiogenesis after myocardial infarction [36,37].

On the other hand, the genes classified in the second cluster were up-regulated after S-MenSCs treatment, such as CXCR6, ACKR2, and BMP6 (among others). In the case of CXCR6 (chemokine receptor for CXCL16), a recent publication has reviewed the contribution of chemokines and their receptors in myocardial infarction [38]. In vivo experiments using CXCR6 knock-out mice have demonstrated that the disruption of the CXCL16/CXCR6 signaling had a cardioprotective effect against ischemia-reperfusion injury [39]. Additionally, CXCR6 knock-out mice demonstrated that CXCR6 could reduce the infarct size by reducing the infiltration of IL17a producing iNKT cells [40]. In the case of ACKR2, this molecule is a scavenging receptor for inflammatory cytokines and was found to be involved in the resolution of acute inflammation [41]. In myocardial infarction, ACKR2 was shown to prevent excessive infiltration of monocytes and neutrophils reducing adverse cardiac remodeling [42]. Based on that, our results may suggest that the increase of CXCR6 and ACKR2 receptors in the infarcted areas of AMI/S-MenSCs could be associated with a chemokine clearance, which may reduce excessive recruitment of inflammatory cells. Regarding the BMP6 release observed in the infarcted areas of animals treated with S-MenSCs*, previous studies have demonstrated that the BMP family is involved in angiogenesis and neovessel formation [43]. Additionally, BMP6 expression was found to be decreased in myocardial infarction and considered as a marker of recurrence [44]. Here, we hypothesize that, as it occurs in ischemia-induced brain damage [45], the increase of BMP6 may reduce ischemia/reperfusion injury by inhibiting apoptotic pathways.

In the cluster of up-regulated genes in animals treated with S-MenSCs*, genes such as TFRC or IGF1R were identified. Although only a few studies have been focused on the relationship between myocardial infarction and TFRC expression, preclinical studies using TFRC knock-out mice have demonstrated that these animals had a loss of cardiac function, cardiomegaly, and mitochondrial anomalies [46]. Therefore, the increase of TFRC in the infarcted areas may demonstrate a therapeutic, or at least palliative effect of these secretomes. On the other hand, IGF1R, through the IGF1-IGF1R system, modulates the proliferation of myocytes after myocardial infarction. Changes in IGF1R can be observed in myocytes shortly after infarction, and their activation has been demonstrated to reduce the apoptosis/necrosis of cells during ischemia/reperfusion injury [47,48].

Once the immune-related genes in the infarcted areas of animals were characterized, we aimed to establish the biological networks between the differentially expressed transcripts and the miRNAs identified in the secretomes. This analysis was performed on the top-ten abundant miRNAs previously identified in S-MenSCs and S-MenSCs*. Interestingly, our results demonstrated that some genes related to the GO category Immune response were found to be targets of various top-ten miRNAs. One of these pathways has been described using preconditioning cells in the treatment of myocardial infarction, where miR-21 is a regulator of STAT3 signaling improving cardiac function parameters [49].

Regardless, the relation between miRNAs in secretomes and the decrease/increase of target genes will require further confirmation. In this sense, to better detect cause–effect signatures, it will be interesting to evaluate miRNA-based therapies in animal models using some of the most relevant miRNAs detected in secretomes.

## 4. Materials and Methods

### 4.1. Study Design

All the experimental procedures involving animals were validated by the Ethics Committee on Animal Experiments of the Jesús Usón Minimally Invasive Surgery Centre, following the recommendations outlined by the local government (Junta de Extremadura), and the EU Directive 2010/63/EU of the European Parliament on the protection of animals used for scientific purposes.

Female Large White pigs (*n* = 12), weighing 36.68 ± 5.18 kg at the beginning of the study, were used for the myocardial infarction model creation. During the procedures, the animals were housed in the animal facility of the Jesús Usón Minimally Invasive Surgery Centre and their final destination was euthanasia. The sacrifice was performed under deep anesthesia through an intravenous administration of 2 mmol/kg of KCl.

**Figure 5:**
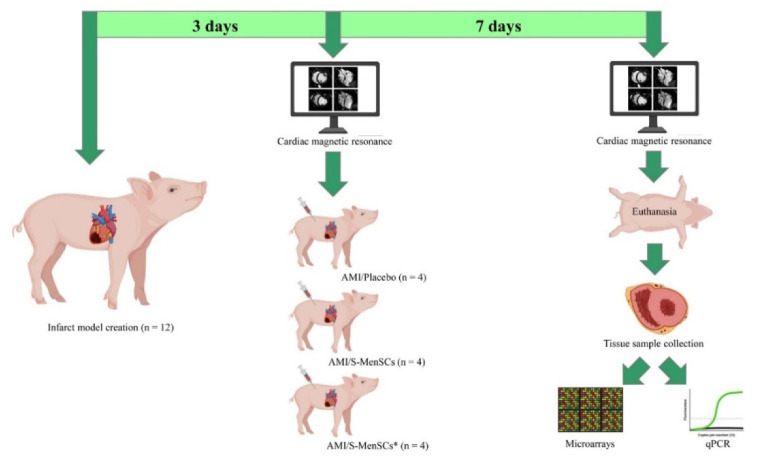
Study design. Three days after infarct model creation, cardiac function parameters were evaluated by cardiac magnetic resonance. Immediately, the animals were divided into three biogroups and subjected to the intrapericardial administration of: AMI/Placebo that received culture medium; AMI/S-MenSCs that received S-MenSCs; and AMI/S-MenSCs* that received S-MenSCs*. 7 days after therapy, the animals were evaluated again by cardiac magnetic resonance immediately before euthanasia. Explanted hearts were sectioned following heart anatomy and the corresponding infarcted samples were collected to perform microarrays and qPCR analysis. Images have been created with BioRender (https://app.biorender.com/, accessed on 17 March 2022). S-MenSCs: secretome from endometrial-derived mesenchymal stromal cells; S-MenSCs*: secretome from endometrial-derived mesenchymal stromal cells primed with IFNγ and TNFα; AMI: acute myocardial infarction.

The animals used for myocardial infarction model creation were randomly distributed into three groups according to the administered treatment: AMI/Placebo (*n* = 4), AMI/S-MenSCs (*n* = 4), and AMI/S-MenSCs* (*n* = 4). The protocol time points between the infarction model creation to the tissue analysis are schematized in Figure 5.

### 4.2. Isolation, Preconditioning, and Characterization of S-MenSCs

This procedure was approved by the Minimally Invasive Surgery Centre Research Ethics Committee (approval number: 017/16) and performed following its ethical guidelines. To participate in the study, the female donors signed an informed consent form.

S-MenSCs were collected from the MenSCs culture medium, then concentrated and characterized according to previously described protocols [16,50]. Briefly, the cells were isolated from the menstrual blood of four healthy women aged up to 40 years. Samples were collected on day 2 or 3 of the menstrual cycle in a menstrual cup, diluted 1:2 in PBS, and centrifuged at 450× *g* for 10 min. The supernatant was discarded, and the pellet was resuspended in Dulbecco’s Modified Eagle’s medium (DMEM) (Merck-MilliporeSigma, Burlington, MA, USA) (containing 10% fetal bovine serum (FBS) (Thermo Fisher Scientific, Waltham, MA, USA), 1% penicillin/streptomycin, and 1% glutamine). Subsequently, cells were cultured in a tissue culture flask (Thermo Fisher Scientific) and expanded at 37 °C and 5% CO_2_. Nonadherent cells were removed after 24 hr. Adherent cells were in vitro expanded up to a confluence of 80%, and passages 5–6 were used for secretome collection.

For phenotypic analysis of MenSCs, the expression levels of CD29, CD44, CD73, CD90, CD105, CD117, CD14, CD20, CD31, CD34, CD45, CD80, and HLA-DR were detected by flow cytometry (BD FACSCalibur, USA). Oil Red O, Alcian Blue, and Alizarin Red S staining (Thermo Fisher Scientific) were performed to evidence adipogenic, chondrogenic and osteogenic differentiation, respectively. Cells were analyzed at passages 3–4.

The cells with confluency up to 80% were primed with 100 ng/mL human IFNγ Recombinant Protein and TNFα (Miltenyi Biotec Inc., Auburn, CA, USA) in DMEM with 10% FBS and 1% penicillin/streptomycin for 3 days. Then, this standard culture medium was replaced by S-MenSCs concentration medium compounds by DMEM with 1% insulin–transferrin–selenium (Thermo Fisher Scientific) after rinsing three times with PBS. After 4 days, the supernatants were collected, centrifuged at 1000× *g* for 10 min and 5000× *g* for 20 min at 4 °C to eliminate dead cells and debris. Supernatants were then filtered using firstly 450 nm pore size sterile cellulose acetate filters, followed by 200 nm pore size filters. Then, 3 kDa MWCO Amicon Ultra devices (Merck-MilliporeSigma) were used to concentrate up to 15 mL filtered supernatants, by centrifugation at 4000× *g* for 60 min at 4 °C. The obtained concentrated supernatants were collected, characterized, and stored at −80 °C for the subsequent analyses. Particle concentration, size profile and phenotype of the vesicles in the S-MenSC and S-MenSC* samples were analyzed by nano-flow cytometry (nFC) as previously described by our group. The miRNome was characterized by Next-Generation Sequencing (NGS) and the datasets are deposited in Sequence Read Archive (SRA) data with the accession number PRJNA664968 [18].

### 4.3. AMI Model Creation and Monitoring

AMI model was generated using a closed chest reperfused myocardial infarction technique, following the procedure previously published by our group [51]. During the procedure, all animals were anesthetized, monitored, and medicated to prevent animal suffering and control protocol deviations. Briefly, access to the left anterior descending coronary artery was achieved with a guiding catheter inserted via the right femoral artery. Coronary occlusion was performed with a percutaneous transluminal coronary angioplasty balloon catheter (typically 3 mm × 10 mm, Ryujin Plus, Terumo) for 90 min.

Cardiac magnetic resonance exams were performed before the administration of secretomes (72 h after myocardial infarction) and at the end of the study (7 days after administration). All studies were conducted with Intera 1.5 T equipment (Philips Medical Systems, Best, The Netherlands) and the image acquisition protocol as previously described [51].

### 4.4. Intrapericardial Administration

Intrapericardial administration was the chosen route for treatment administration. Animals were anesthetized and monitored during the procedure as previously described [12]. The access route to the pericardium was by a lateral mini-thoracotomy with an incision on the fourth or fifth intercostal space to expose the pericardial sac. After incision, and with the help of a rib retractor, delicate traction is exerted in the pericardium to allow the introduction of an Abbocath-T 20G catheter (Hospira, Lake Forest, IL, USA) within it. Treatment was delivered slowly in the pericardial space while checking for the appearance of arrhythmias (Figure S1).

Animals received the treatment in a total volume of 5 mL. AMI/Placebo group (*n* = 4) received concentration medium (DMEM + 1% ITS), AMI/S-MenSCs group (*n* = 4) received a total of 9.16 mg S-MenSCs proteins per animal, and AMI/S-MenSCs* group (*n* = 4) received a total of 9.16 mg S-MenSCs* proteins per animal, both diluted in concentration medium. Immediately after administration, the incision was sutured in layers and the animals were allowed to recover.

### 4.5. Tissue Collection and RNA Isolation

**Figure 6:**
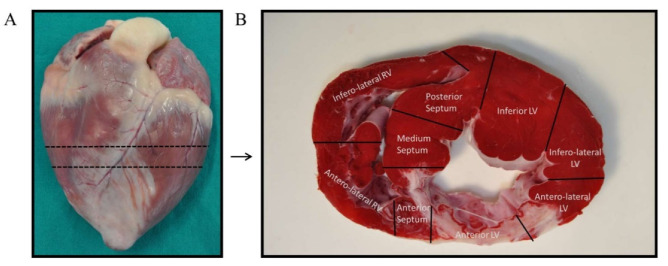
Tissue samples collection. (**A**) The intact heart was sectioned in its short-axis plane at the mid-ventricle. (**B**) The mid-ventricular slice was divided into nine sections following heart anatomy: four from the left ventricular walls (anterior, inferior, anterolateral, and inferolateral), three from the interventricular septum (anterior, medium, and posterior), and two from the right ventricular walls (anterolateral and inferolateral). The anterior left ventricular sample was selected for the experiments of this study.

Previous to tissue collection, animals were euthanized by an intravenous lethal dose (2 mmol/kg) of KCl while under deep anesthesia. Immediately after, hearts were extracted by thoracotomy and washed with PBS to remove residual blood. Each intact heart was divided into short-axis ices, of which, a mid-ventricular section was selected to obtain tissue samples (Figure 6A). Following the criteria established by Galati G. [52], the mid-ventricular section was, in turn, segmented in nine samples depending on the heart anatomy: four from the left ventricular walls (anterior, inferior, anterolateral, and inferolateral), three from the interventricular septum (anterior, medium, and posterior), and two from the right ventricular walls (anterolateral and inferolateral) (Figure 6B). Sample from the anterior left ventricular wall (infarct area) was selected for RNA extraction. This tissue was minced and collected in Allprotect Tissue Reagent (Qiagen, Hilden, Germany) at −20 °C to preserve RNA integrity until the day of isolation.

RNA was isolated with PureLink RNA Mini Kit (Thermo Fisher Scientific), using TRIzol reagent. RNA concentration and quality were determined by Agilent 2100 Bioanalyzer (Agilent Technologies, Santa Clara, CA, USA). All samples were optimal for microarray experiments and qPCR.

### 4.6. Microarray Expression Analysis

RNA from tissue samples was reverse-transcribed, labeled, and hybridized using Affymetrix GeneChip WT Pico Reagent Kit (Thermo Fisher Scientific) according to the user guide. Data generation was assessed by scanning GeneChip Porcine Gene 1.1 ST Array Plate in a Gene Titan Affymetrix microarray platform. Files were pre-processed for background correction and normalization using Robust Multichip Average (RMA) methodology [53] implemented in the rma function of Bioconductor package oligo [54] resulting in 27,558 features from 12 samples for downstream analysis. Array probes were annotated to genes according to PorGene-1_1-st-v1 annotation, Release 36 provided by the manufacturer.

Multiple comparisons were performed, and *p* values were calculated. The inter- and intra-variability of study groups, as well as limitations in terms of sample size, made it difficult to reach a statistically significant difference using the False Discovery Rate (FDR) approach. Transcripts with *p* values < 0.05 (without FDR optimization) were considered differentially expressed and selected for subsequent analysis and validations by qPCR. Additionally, this analysis was also restricted to transcripts clustered in the *Immune response* (GO:0006955) term, included in Biological Process (GO BP) from Gene Ontology Resource and filtered by *Sus scrofa* organism. Finally, these selected genes were clustered in a heatmap and a principal component analysis (PCA) using the ClustVis online tool [55]. The microarray data have been deposited in Gene Expression Omnibus (GEO) database with the GEO Submission GSE171740.

### 4.7. Networks of miRNA and Gene Interactions

S-MenSCs and S-MenSCs* miRNome data were obtained from Sequence Read Archive (SRA) database with the accession number 215 PRJNA664968 (Table S1). The top-ten of more expressed miRNAs for both secretomes were selected and gene-targeted analysis was performed using miRTargetLink web tool (https://ccb-web.cs.uni-saarland.de/mirtargetlink/, accessed on 22 February 2022). Only genes with strong experimental evidence were considered. These genes were translated into their orthologue in pig using g:Profiler [56] based on the information retrieved from the Ensembl database. Output data were compared with differentially expressed genes (DEGs) in pig heart tissue. The network between the top-ten expressed miRNAs in secretomes and the DEGs in pig heart tissue was represented using Cytoscape 3.7.2. software [57].

### 4.8. qPCR Analysis for Validation of Pre-Selected Transcripts

According to the bibliography, nine transcripts were selected for validation of transcriptomic results by qPCR. For each sample, 1 µg of isolated RNA was used for cDNA synthesis using iScript Reverse Transcription Supermix (BioRad, Hercules, CA, USA), according to the manufacturer’s instructions. Following retrotranscription, amplification was performed with commercial TaqMan Gene Expression Assays probes (Thermo Fisher Scientific) (Table S2) and TaqMan Fast Advanced Master Mix (Thermo Fisher Scientific), following the protocol provided by the manufacturer. Templates were amplified and recorded in a QuantStudio 3 Real-Time PCR System (Thermo Fisher Scientific). qPCR products were analyzed in the Thermo Fisher Cloud, normalized using the RPS3 gene, and quantified using the 2−ΔΔCt method. RPS3 gene was chosen as an endogenous gene because of its stable expression in microarray results with a low coefficient of variation (CV) and high expression levels [58].

### 4.9. Statistical Analysis

Cardiac magnetic resonance data were statistically analyzed with GraphPad Prism 6 software. Differences between biogroups were calculated using the Kruskal–Wallis and Mann–Whitney U tests, and intragroup comparisons were determined with the Wilcoxon paired samples test.

Modeling for biogroups, linear regression for DEG analysis from normalized expression arrays were calculated and refined after model fitting, corrected for multiple testing by Benjamini–Hochberg using of Bioconductor R package Limma [59].

Normal distribution of qPCR results was assessed using the Shapiro–Wilk test, and correlation between qPCR and transcriptomics results was calculated with a Pearson coefficient correlation. All *p* values < 0.05 were considered statistically significant.

## 5. Conclusions

In summary, the methodology of this study allowed us to evaluate the cardiac function of treated and untreated animals and to identify thousands of transcripts expressed in the infarcted areas of a porcine model. Here, we identified a panel of immune-related genes that were modified in the infarcted myocardium using S-MenSCs and S-MenSCs* as therapeutic agents. Finally, we found that some of the DEGs were targets for the top abundant miRNAs detected in the secretome of MenSCs. Altogether, our transcriptomic analysis provides some clues to fathom the role of secretomes from MSCs as therapeutic agents for myocardial infarction.

## Data Availability

The data presented in this study are openly available in Gene Expression Omnibus (GEO) database with the GEO Submission GSE171740 (https://www.ncbi.nlm.nih.gov/geo/query/acc.cgi?acc=GSE171740, accessed on 22 February 2022).

## Supplementary Materials

The following supporting information can be downloaded at: https://www.mdpi.com/article/10.3390/biomedicines10051117/s1, Figure S1: Intrapericardial administration of the therapy, Table S1: List of top-ten abundant miRNAs in secretomes from MenSCs cultured under standard culture conditions (A) and prime with IFNγ/TNFα (B). Counts per million in a Next-Generation Sequencing analysis, Table S2: Assays ID of commercial TaqMan Assays.
