# Supplementary material for: Intrapericardial Administration of Secretomes from Menstrual Blood-Derived Mesenchymal Stromal Cells: Effects on Immune-Related Genes in a Porcine Model of Myocardial Infarction
Source: Biomedicines. 2022 May 11;10(5):1117. doi: 10.3390/biomedicines10051117 (PMC9138214; doi:10.3390/biomedicines10051117)
Supplement: Supplementary file 1 [file biomedicines-10-01117-s001.zip › biomedicines-1686925-supplementary.pdf]

Supplementary Materials

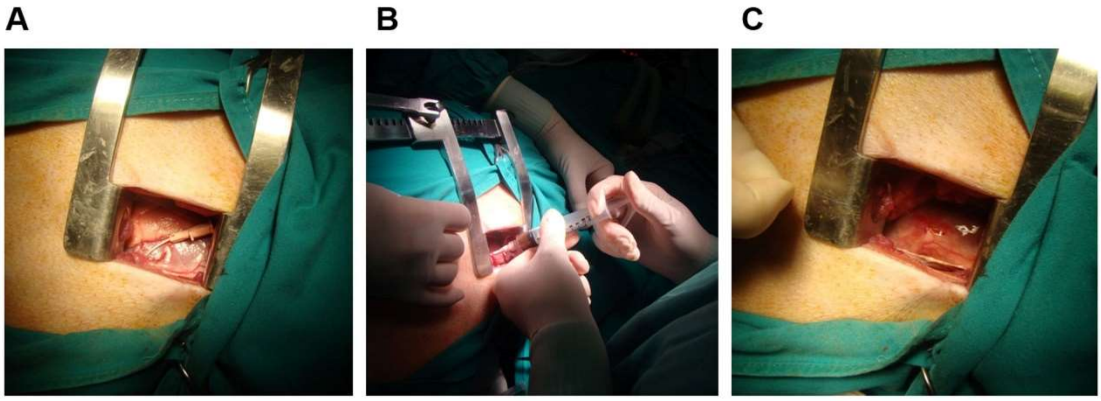

Figure S1. Intrapericardial administration of the therapy. (A) An Abbocath® was inserted into the pericardial space via a mini-thoracotomy. (B) The injection was performed slowly. (C) When needed, a 0/6 polypropylene suture was applied to the pericardium.

Supplementary Materials

Table S1. List of top-ten abundant miRNAs in secretomes from MenSCs cultured under standard culture conditions (A) and prime with IFNγ/TNFα (B). Counts per million in a Next-Generation Sequencing analysis.

A

miRNAs	MenSC01	MenSC02	MenSC03	Mean	SD
hsa-miR-143-3p	126452	69402	153649	116501	42996
hsa-miR-12136	50103	89693	70610	70136	19799
hsa-let-7b-5p	41554	113086	51489	68710	38750
hsa-miR-199a-3p	53483	60355	47802	53880	6286
hsa-miR-21-5p	43145	24039	46709	37964	12191
hsa-let-7a-5p	30420	47173	36056	37883	8525
hsa-miR-29a-3p	18093	36704	37422	30740	10958
hsa-let-7f-5p	29426	24943	27452	27274	2246
hsa-let-7i-5p	22467	15767	23081	20439	4057
hsa-miR-27a-5p	18491	4653	26496	16546	11051

B

miRNAs	MenSC*01	MenSC*02	MenSC*03	Mean	SD
hsa-miR-199a-3p	143125	174179	139845	152383	18947
hsa-miR-143-3p	118695	47687	129049	98477	44289
hsa-miR-29a-3p	80038	74413	81931	78794	3910
hsa-miR-221-3p	13448	143693	29323	62155	71059
hsa-miR-21-5p	106025	24136	53797	61320	41459
hsa-let-7i-5p	71519	61377	38244	57047	17055
hsa-miR-27b-3p	25598	66848	37878	43441	21180
hsa-miR-27a-3p	89853	22380	15828	42687	40978
hsa-let-7b-5p	21015	65317	39021	41784	22280
hsa-let-7f-5p	36365	37893	27082	33780	5851

Table S2. Assays ID of commercial TaqMan Gene Expression Assays.

Gene Symbol	Assay ID
BMP6	Ss04245858_m1
CXCR6	Ss03818556_s1
ACKR2	Ss04953541_s1
LIF	Ss03391458_m1
STAT3	Ss03388426_m1
APOE	Ss03394681_m1
TFRC	Ss03391240_m1
THBS1	Ss03373620_m1
BCL6	Ss04955003_m1
CDC42	Ss03388722_m1
IGF1R	Ss03394281_m1
RPS3	Ss01037177_g1
